# Urinary exosomes: a promising biomarker of drug-induced nephrotoxicity

**DOI:** 10.3389/fmed.2023.1251839

**Published:** 2023-09-22

**Authors:** Zunzhen Zhou, Dailiang Zhang, Yongjing Wang, Chongzhi Liu, Limei Wang, Yi Yuan, Xiaodan Xu, Yuan Jiang

**Affiliations:** ^1^Clinical Medical College, The First Affiliated Hospital of Chengdu Medical College, Chengdu, Sichuan, China; ^2^Department of Rehabilitation Medicine, The Third Affiliated Hospital of Chengdu Medical College, Chengdu, Sichuan, China; ^3^Orthopedic Department, Dazhou Integrated TCM and Western Medicine Hospital, Dazhou Second People’s Hospital, Dazhou, China

**Keywords:** drug-induced nephrotoxicity, acute kidney injury, biomarkers, urinary exosomes, kidney disease

## Abstract

Drug-induced nephrotoxicity (DIN) is a big concern for clinical medication, but the clinical use of certain nephrotoxic drugs is still inevitable. Current testing methods make it hard to detect early renal injury accurately. In addition to understanding the pathogenesis and risk factors of drug-induced nephrotoxicity, it is crucial to identify specific renal injury biomarkers for early detection of DIN. Urine is an ideal sample source for biomarkers related to kidney disease, and urinary exosomes have great potential as biomarkers for predicting DIN, which has attracted the attention of many scholars. In the present paper, we will first introduce the mechanism of DIN and the biogenesis of urinary exosomes. Finally, we will discuss the changes in urinary exosomes in DIN and compare them with other predictive indicators to enrich and boost the development of biomarkers of DIN.

## Introduction

The kidney is an essential organ for drug metabolism and excretion. Due to its unique structure and function, the kidney is particularly susceptible to drug damage and is the target organ of drug toxicity (1). Drug-induced nephrotoxicity (DIN) refers to renal injury caused directly or indirectly by drugs and is one of the most common side effects in clinical drug therapy. DIN is the main causative factor of acute kidney injury (AKI), chronic kidney disease (CKD), acute renal failure (ARF), and end-stage renal disease (ESRD) (2, 3). About 14–26% of adults and 16% of children may experience DIN during clinical medication, especially in the elderly, who may have multiple chronic diseases requiring multiple drug treatments (4). Drug-related renal dysfunction is usually reversible, and patients’ renal function will improve after drug withdrawal. However, DIN may cause damage to different nephron segments (e.g., glomeruli and tubules) and irreversible structural damage to renal tissue without early detection and timely treatment. Some scholars proposed to classify DIN into acute phase (1–7 days), subacute phase (8–90 days), and chronic phase (duration>90 days, the development of chronic kidney disease) based on the time course and period of renal dysfunction (5). In addition to drug-related elements, other risk factors, including patients, age, gender, diet, and renal function, can affect the incidence of DIN. Clinicians should consider the following conditions when diagnosing DIN (6): (a) The time of exposure to drugs must be at least 24 h and must be before the renal injury; (b) Suspicious drugs that cause kidney damage may have biological evidence; (c) Assess suspicious drugs and associated risks and consider whether to come into contact with other nephrotoxic drugs; (d) The strength of the association between suspected drugs and injury should be based on drug exposure and treatment time. Clinicians often evaluate drug toxicity depending on the biomarkers of renal injury, such as blood urea nitrogen (BUN), serum creatinine, estimated glomerular filtration rate (eGFR), and urinary albumin. But these traditional renal function testing methods are difficult to detect early renal injury and lack sensitivity and reliability in predicting drug nephrotoxicity (7). The selection of biomarkers is critical in the evaluation of DIN. The ideal biomarkers need not only reflect the degree of renal injury but also evaluate renal function and specific drug toxicity. Urine is an excellent sample source for biomarkers related to kidney disease. In recent years, some nephrotoxic biomarkers from urine have been applied in preclinical and clinical safety assessments of drugs, showing good prospects. In particular, urinary exosomes have attracted the attention of us.

Urine samples are easy to collect but are susceptible to external environmental factors that can affect the urinalysis results. For example, free urinary RNA is easily degraded by urinary RNase, while urinary protein is easily contaminated. Urinary exosomes are a kind of extracellular vesicles (VEs) with 40–200 nm size secreted by various cells in the urinary system and released into the urine (8). In general, the circulating exosomes are hard to enter urine through the glomerulus under physiological conditions, so the source of urinary exosomes is limited to the kidney. Urinary exosomes can be easily obtained and stably stored from urine. In particular, their contents include bioactive molecules such as proteins and nucleic acids derived from donor cells that can reflect the state of the kidney under physiological and pathological conditions on time (9–11). The complete membrane structures surrounding the urinary exosomes keep their contents from the urinary environment. They can protect their RNAs from the degradation of RNA enzymes and avoid the contamination of exosomal protein by other protein molecules in the urine. Therefore, urinary exosomes may carry biomarkers of renal dysfunction and structural injury and have unique advantages in the early diagnosis of renal diseases (12, 13).

Previous studies have observed changes in urinary exosomes during drug-induced nephrotoxicity, indicating that drug toxicity will affect the biogenesis of urinary exosomes. The pathogenesis of DIN may determine the correlation between changes in urinary exosomal contents and DIN. In the present paper, we will first introduce the mechanism of DIN and the biogenesis of urinary exosomes. Then we will discuss the changes in urinary exosomes in DIN and compare them with other predictive indicators to enrich and boost the development of biomarkers of DIN.

## Mechanism of DIN

In 2015, Mehta et al. proposed four phenotypes of renal diseases caused by DIN in the expert consensus of phenotypic standardization of drug-induced nephropathy: AKI, glomerular disorder, nephrolithiasis/crystalluria, and renal tubular disorder (5). The clinical manifestations of these subtypes are related to the mechanisms of DIN leading to different renal injury patterns. During drug clearance, renal tubular cells and the surrounding matrix are exposed to drugs through free surface contact and cellular uptake or cell transport through the basal lateral circulation. Thus, the mechanisms of DIN are complicated and involve various aspects, including drug-related toxic effects, alteration in glomerular hemodynamics, Inflammatory immune response, crystallization-induced tubular obstruction, rhabdomyolysis, and thrombotic microangiopathy (TMA) (3).

Drug-related toxic effects: the excretion of drugs is mainly through urine. The kidney can concentrate urine leading to high concentrations of drugs or their metabolite solutions in renal tubules that may damage the cell membrane of renal tubules and cause renal tubular transport dysfunction (14). Some common medications include aminoglycosides (AGs), platinums, amphotericin B, and colistin (15). Taking AGs as an example, AGs include gentamicin, tobramycin, amikacin, netilmicin, neomycin, and streptomycin, which are widely used clinically to treat bacterial infections. But AG-induced nephrotoxicity can induce cytotoxicity to renal tubular cells and a decrease in glomerular filtration and renal blood flow, and its incidence can reach 10–25% (16–18). AGs are polycationic drugs and can bind to megalin/cubulin receptors. Then AGs enter cells through endocytosis from the apical and basal lateral surfaces and accumulate in lysosomes, Golgi bodies, endoplasmic reticulum (ER), and even mitochondria (3, 14). Therefore, AGs can damage lysosomes to activate proteases or directly stimulate mitochondria to produce ROS to damage cells, leading to apoptosis or necrosis in epithelial cells of renal tubules (19). AGs can also increase intracellular calcium levels to active proteases leading to apoptosis and inducing contraction of mesangial smooth muscle to reduce renal blood flow and glomerular filtration rate (GFR) (20). In addition, the accumulation of AGs in cells also leads to phospholipidosis. Phospholipidosis is a disorder of phospholipid metabolism that causes lipotoxicity to exacerbate renal toxicity (21–23).

Alteration in glomerular hemodynamics: Glomerular filtration is closely related to the arterial blood volume and vascular resistance of afferent and efferent arterioles in the glomerulus. The relaxation of the afferent arterioles and the contraction of the efferent arterioles also are contributed to the maintenance and regulation of glomerular pressure. Some studies have found that prostaglandins and angiotensin II regulate the relaxation or contraction of the afferent arterioles and efferent arterioles, respectively (24). Some drugs may significantly influence the prostaglandins and angiotensin II levels, such as anti-prostaglandin activities drugs (e.g., NSAIDs) or the drugs affecting the renin-angiotensin-aldosterone system (e.g., angiotensin-converting enzyme inhibitors-ACEI and angiotensin receptor blockers-ARBs). The effects of ACEI and ARB on renal hemodynamics cause a reversible decrease in GFR without any renal parenchymal damage, but excessive or long-term use can lead to severe contraction of renal blood vessels and a decline in renal blood volume, which induce changes in renal hemodynamics and glomerular filtration rate ultimately causing AKI. The inhibition effect of NSAIDs on cyclooxygenase (COX) and prostaglandin synthesis will lead to vasodilation dysfunction, water-sodium retention, and intraglomerular hypertension (25). Some scholars suggested that concurrent long-term use of ACEI/ARB and NSAIDs may cause the complex interplay among their effects and bring a double whammy for glomerular, resulting in an alteration in glomerular hemodynamics, seriously decreased glomerular filtration and higher risks of nephrotoxicity (26, 27).

Inflammatory immune response: some drugs can cause inflammation and immune reactions in the glomerulus, tubular cells, and stroma, such as NSAIDs, proton pump inhibitors, *β*- Lactam drugs, and antiviral drugs. Patients may even experience typical symptoms of hypersensitivity after medication, such as fever, rash, and eosinophilia (28). Drugs or their metabolites can make a variety of antibodies to react with the antigen components of the glomerular basement membrane to form immune complexes (29). Drugs or their metabolites can produce different kinds of antibodies to react with the antigen components of the glomerular basement membrane to form immune complexes. The immune complexes accumulate in the glomerulus to activate various circulating immune cells, leading to glomerular injury, or enter the interstitial microcirculation to bind the basement membrane of the renal tubules to induce interstitial inflammation. In addition, immune complexes can activate complement pathways to regulate the downstream inflammatory cascades, promoting leukocyte infiltration and worsening kidney damage.

Crystallization-induced tubular obstruction: the poorly soluble drugs and their metabolites can form crystals in the tubular fluid that induce crystal nephropathy. Some common medications include sulphonamides, methotrexate, indinavir, acyclovir, atazanavir, sulfadiazine, ciprofloxacin, aluminum hydroxide, and amoxicillin (30–32). In addition to the acid dissociation constant (pKa) and dosage of the poorly soluble drugs, urinary flow rate and urine pH are important influencing factors of crystals. The decrease in urinary flow rate contributes to the deposition and retention of drugs and their metabolite crystals in the renal tubules (33). Urine pH below 5.5 will increase intratubular crystal deposition of sulfadiazine, methotrexate, and triamterene, while urine pH above 6.0 will increase intratubular crystal deposition of indinavir, atazanavir, and ciprofloxacin (34–36). The crystals will block renal tubular blood flow in the distal renal tubular lumen, leading to tubular injury and a decline in GFR. They can also bind to the basement membrane of renal tubules to trigger peripheral interstitial inflammatory reactions, leading to acute interstitial nephritis (AIN). Tubular injury and AIN both contribute to the development of AKI (3).

Rhabdomyolysis: Rhabdomyolysis is caused by the decomposition and necrosis of muscle tissue and the release of intracellular content into the bloodstream. Its clinical syndromes include severe muscle pain, weakness, and myoglobinuria (37, 38). AKI is one of the most dangerous complications of rhabdomyolysis (39). Skeletal muscle injury can lead to the release of a large amount of myoglobin, but myoglobin as a toxin can cause kidney dysfunction. Skeletal muscle injury can lead to the release of a large amount of myoglobin, but myoglobin as a toxin can cause kidney dysfunction. Myoglobin activates the complement bypass pathway and lectin pathway and causes an oxidative stress reaction in the process of glomerular filtration, causing damage to renal tubular cells and their organelles (39, 40). In addition, myoglobin can also lead to nephrotoxicity by causing renal vasoconstriction and tubular formation (39). In addition, myoglobin can cause renal vasoconstriction to affect glomerular hemodynamics (41). For example, statins are currently the most clearly defined drugs that cause rhabdomyolysis, and their most serious adverse reaction is muscle toxicity. The average incidence rate of atorvastatin, pravastatin, or simvastatin monotherapy was 0.44 per 10,000 person-years. Benzodiazepines, antidepressants, antihistamines, anesthetic drugs, and ethanol may make patients susceptible to rhabdomyolysis (42–44). In addition, excessive abuse of illegal drugs such as cocaine can also lead to rhabdomyolysis and microvascular thrombosis (45, 46).

Thrombotic microangiopathy (TMA): TMA is the thrombosis and ischemic injury of capillaries and arterioles in renal parenchyma caused by the severe damage of vascular endothelial cells (47). TMA is characterized by thrombotic thrombocytopenic purpura (TTP) and hemolytic uremic syndrome (HUS) (48, 49). Some drugs can induce immune-mediated impairment to affect the aggregation and consumption of platelets or direct toxicity to vascular endothelial cells, leading to thrombocytopenia, microvascular thrombosis, and mechanical hemolysis, for example, antiplatelet agents (e.g., clopidogrel and ticlopidine), anti-infective agents (e.g., vancomycin, sulfamethoxazole, and metronidazole), antineoplastic agents (e.g., mitomycin C and gemcitabine), and quinine (50–53).

## Biogenesis of urinary exosomes

**Figure 1:**
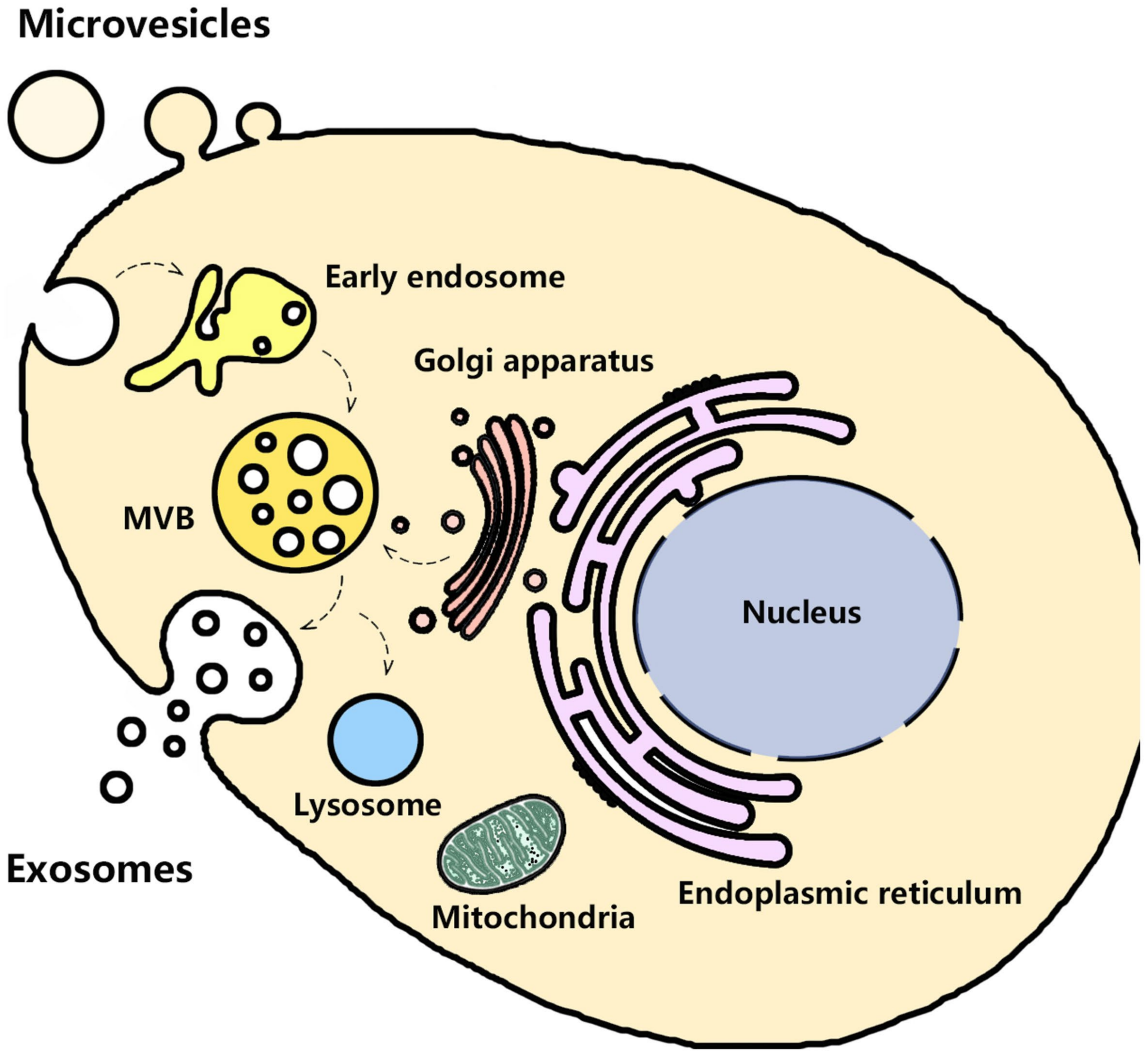
Schematic representation of exosome biogenesis.

Urine contains exosomes and other EVs (e.g., microvesicles and apoptotic bodies) that originate from several parts of the urogenital tract, such as kidneys, bladder, prostate (in males), and even uterovaginal tract (in females). In 1986 Wiggins and colleagues first observed these membrane-bound vesicles by transmission electron microscopy (TEM) (54). Until 2004, Pisitkun and colleagues harvested urinary exosomes by ultracentrifugation. They provided a complete characterization of urinary exosomes and promoted follow-up research on urinary exosomes (55). Puhka and colleagues found that the isolated urinary exosomes by ultracentrifugation were cup-shaped and enriched with typical markers CD9, TSG101, CD59, and CD63 (56). Urinary exosomes from the different parts of the urogenital system possess their characteristic proteins. For instance, glomerular podocyte-derived exosomes are enriched in podocin, podocalyxin, or nephrin. Proximal tubular cells-derived exosomes have megalin, cubilin, aminopeptidase, or aquaporin-1 (AQP1) (57). But urinary exosomes have the same biogenesis pathways that start from the inward budding of the cellular plasma membrane and then mature into late endosomes. Late endosomes bud into intraluminal vesicles (ILVs) and multivesicular bodies (MVBs) and encapsulate nucleic acids, proteins, and other bioactive molecules. After fusion with the cell membrane, MVBs are released into the intercellular space as exosomes, while the other MVBs are degraded after fusion with lysosomes, as shown in Figure 1 (58, 59). The isolated urinary exosomes in urine can be captured by neighboring or distant cells to modulate cell function, indicating that urinary exosomes are involved in the pathophysiological process of kidney diseases (60–62). Previous studies have shown that the quantity and contents of urinary exosomes significantly changed in kidney diseases, which may provide valuable information to evaluate the progress. Urinary exosomes can protect their molecular cargoes from the impact and contamination of other substances in the urine, so they are suitable as a biomarker for glomerular and tubular damage (63). During the biogenesis processes, the endoplasmic reticulum-Golgi apparatus route (ER-Golgi pathway) and mitochondrial-lysosomal axis contributed to the sorting of some proteins and the transport of vesicles to the cell membrane for cell membrane fusion and secretion of exosomes (64–66). The composition and secretion of exosomes are mainly dependent on the involvement of endosomal sorting complex required for transport (ESCRT)-dependent pathway, ESCRT independent pathway, Rab-GTPase family, SNARE family, lipid raft, syndecan-1, and four-transmembrane domain proteins (67). Hence, the production of exosomes may be affected by some organelles, and the mechanism of sorting exosomal contents is complex. Considering that the mechanism of DIN involves various aspects, the changes in urinary exosomes in the DIN need further discussion.

## Urinary exosomes and DIN

Based on current knowledge of the biogenesis of urinary exosomes, the structural and functional integrity of some organelles (e.g., lysosomes, endoplasmic reticulum, and mitochondria) and the intracellular and extracellular environment (e.g., Ca^2+^, ROS, and hypoxia) are closely related to the biogenesis pathways of exosomes. Lysosomes, endoplasmic reticulum, and mitochondria organize into a dynamic network through some crosstalk. Defects in one or two organelles can cause damage to the other, resulting in a change in Intracellular homeostasis (65). The toxic effect of drugs will damage the above organelle and destroy their structural integrity and biological function directly or indirectly. Lysosome dysfunction will affect the degradation of MVBs, increasing exosome production, and also affect the regulation of transient receptor potential mucolipin-1 (TRPML-1) on the release of lysosomal calcium ions into the cytoplasm to be involved in the biogenesis of exosomes by change the intracytoplasmic Ca^2+^ signal (68–70). In addition, lysosomal alkalization under lipotoxic conditions may induce mitochondrial dysfunction (73). Some scholars suggested that the dysfunctional mitochondrial-lysosomal axis causes abnormal exosome trafficking (90). Mitochondrial dysfunction not only affects the function of the respiratory chain, leading to changes in ATP and ROS production but stimulates oxidative stress and inflammatory responses that can affect the yield of exosomes (76, 79, 94, 108). The endoplasmic reticulum is a critical intracellular calcium pool. It maintains the stability of intracellular calcium and regulates many processes by its connection with mitochondria, including lipid transport, mitochondrial morphology, cell death, immune response, and autophagy formation (65). Once calcium homeostasis of the endoplasmic reticulum is disturbed, calcium ions are released into the cytoplasm to affect the production of exosomes and further induce endoplasmic reticulum stress contributing to DIN (87). In addition, some drugs can alter glomerular hemodynamics or crystallization-induced hypoperfusion to generate hypoxia conditions in the kidney. It will affect the production of exosomes and exosomal content (e.g., miRNA, mRNA, and protein) (86).

In recent years, some research results have found a close correlation between changes in urinary exosomal miRNA and protein levels and AKI, which indicates that urinary exosomes may serve as biomarkers for early prediction of DIN, as shown in Table 1. Sonoda and colleagues found that urinary exosomal miR-16-5p, miR-24-3p, and miR-200c-3p increased in AKI rats on the first day after ischemia–reperfusion injury (I/R). Those exosomal miRNAs changed their target mRNA expression in the renal medulla, suggesting that the change in urinary exosomal RNAs could mirror the cellular gene expression in kidneys and the progression of AKI (98). Yu and colleagues found that miR-20a-5p was abundant in hypoxia-induced tubular exosomes (Hy-EXOs) by using exosome miRNA sequencing, and miR-20a-5p may provide a protective effect on tubular injury by inhibition of mitochondrial damage and apoptosis in acute tubular injury (107).

**Table 1:** The changes in urinary exosomal miRNA and protein levels in AKI.

Exosomal miRNA/protein	Type of mode	Expression level	References
miR-16-5p, miR-24-3p, miR-200c-3p	I/R-induced AKI rats	Up-regulated	(98)
miR-20a-5p	Renal tubular epithelial cells	Up-regulated	(107)
Fetuin-A	Cisplatin-induced AKI rats, AKI patients	Up-regulated	(110)
Activating transcription factor 3	I/R-induced AKI rats, cisplatin-induced AKI rats, AKI patients	Up-regulated	(109)
C3 complement, C4 complement, galectin-3-binding protein, fibrinogen, alpha-2 macroglobulin, immunoglobulin heavy constant mu, serotransferrin	Vancomycin-induced AKI patients	Up-regulated	(74)
Aquaporin-1	I/R rats	Down-regulated	(99)

Zhou and colleagues evaluated the protein profile of urinary exosomes in the rats injected with cisplatin by a two-dimensional difference in gel electrophoresis and mass spectrometry. They found that exosomal Fetuin-A had increased significantly before increased serum creatinine and tubule damage. Compared to the patients without AKI, the urinary exosomal Fetuin-A also increased in three ICU patients with AKI (110). In subsequent studies, they also found increased protein expressions of activating transcription factor 3 (ATF3) in urinary exosomes at the early renal injury state in both I/R- and cisplatin-induced AKI rats. And these changes in urinary exosomal ATF3 can be observed two days before the increase in serum creatinine and histological changes. Then they also observed similar trends in patients with AKI that ATF3 was continuously present in urinary exosomes at times earlier than the increase in serum creatinine (109). The above results suggested that urinary exosomal Fetuin-A and ATF3 may be novel biomarker candidates for AKI. Awdishu and colleagues collected the urinary samples of patients with vancomycin-induced acute kidney injury (V-AKI) and healthy controls to determine the protein contents of urinary exosomes by label-free liquid chromatography-mass spectrometry (LC/MS). They found 251 proteins were dysregulated in V-AKI patients, especially C3 complement, C4 complement, galectin-3-binding protein, fibrinogen, alpha-2 macroglobulin, immunoglobulin heavy constant mu, and serotransferrin were significantly associated with V-AKI. Those proteins may be predominantly involved in the inflammatory and coagulation pathways, revealing immune responses to direct toxicity of vancomycin in kidney tubular cells, and potentially serve as biomarkers along the continuum of DIN (74). Of course, the expression of some exosomal proteins will significantly decrease in the early stages of acute kidney injury, indicating these proteins can be the urinary biomarkers. Sonoda and colleagues found that the urinary exosomal aquaporin-1 (AQP1) expression level of I/R rats had decreased after 6 h of renal I/R, and the level continued to be low even over 96 h after I/R (99).

## Urinary exosomes and other biomarkers of DIN

Traditional biomarkers BUN and serum creatinine are not very specific or sensitive in assessing renal dysfunction, as they increase only after renal injury (83). Other indicators like urinalysis, electrolytes, and urine sediment are also susceptible to the toxicity of other organs. Recently, scholars have suggested that biomarkers from blood or urine may be better than BUN and serum creatinine for evaluating DIN (85). The US FDA and the European Medicines Agency have approved seven nephrotoxic biomarkers for preclinical and clinical safety assessments of drugs. These biomarkers include clusterin, kidney injury molecule-1 (KIM-1), trefoil factor 3 (TFF-3), urinary albumin, urinary total protein (uTP), *β* 2-microglobulin (B2M), and cystatin C (CysC) (81, 88). Clusterin, KIM-1, TFF-3, and urinary albumin are used to evaluate drug-induced renal tubular damage. uTP, B2M, and CysC are used to indicate drug-induced glomerular injury or renal tubular reabsorption disorder. In 2019, Griffin and colleagues introduced the research progress in the above seven biomarkers and other biomarkers in detail in their review, such as interleukin-18 (IL-18), neutrophil gelatinase-related lipid carrier protein (NGAL), reticulin-1, fatty acid binding protein (FABP), TIMP2, IGFBP7, and urinary exosomes. They suggested that these biomarkers possess the potential for the prediction and diagnosis of DIN, but the changes in these biomarkers may also associated with other kidney diseases or non-kidney diseases, indicating a lack of specificity of these biomarkers for DIN. The correlation between rises in these biomarkers and the development of clinically significant AKI is unclear, and some data are mainly derived from animal models or human clinical data is insufficient (e.g., TFF3, urinary albumin, Clusterin, and NGAL), so further studies are still needed, especially in human subjects (1). In recent years, scholars have also been exploring other new biomarkers, such as mitochondrial DNA (mtDNA), which is closely associated with the loss of kidney function. The levels of mtDNA in peripheral serum and urine also reflect the state of renal injury (84). So far, the above biomarkers still have not replaced serum BUN and serum creatinine in clinical applications. Encouragingly, there is a growing recognition of the potential role of exosomes in the occurrence, development, and prognosis of various diseases in related research on exosomes. Exosomes as biomarkers have more investigation and application values in malignant tumors, brain diseases, cardiovascular diseases, etc. (104–106). The cells of the kidney can also secrete and release exosomes into the urine. The urinary exosomal mRNA and proteins may be upregulated or downregulated after exposure to medication, and the changes happen at the early renal injury state and even earlier than serum creatinine and histological changes, suggesting they will be adequate to detect nephrotoxicity and subsequent renal reactions (100). Urinary exosomes have some advantages among other biomarkers of DIN (e.g., KIM-1, TFF-3, clusterin, etc.) due to their characteristics, such as stable structure, less susceptibility to urinary factors, early changes of content, and non-invasive acquisition.

## Methodology of urine exosomes

Soluble biomarkers in urine are easier to obtain, but sample and biological complexity may lead to some limitations in gaining valuable information efficiently. In contrast, urine exosomes around a lipid bilayer protect their content and offer the specific bio information of DIN with the ease and safety of urine collection for noninvasive tests. The Isolation and purification of exosomes from urine samples are essential to avoid potential differences in different quantitative and compositional analysis protocols. Street and colleagues introduced each step of the protocols for detecting urinary exosomal biomarkers, from ultracentrifugation/isolation/purification, quantitation, composition/constituent analysis, normalization, storage and processing, and data analysis in their published review (101). In short (a) the collected urine samples are usually stored under freezing conditions and subjected to pre-treatment before separation to remove the interference from various urinary tract epithelial cells, cell fragments, and urinary proteins. (b) The isolated urine exosomes can be harvested through separation techniques such as ultracentrifugation, filtration, precipitation, affinity purification, and microfluidics. (c) The harvested urine exosomes need further characterization and quantification. Exosomes’ particle size, quantity, and characteristic proteins can be detected by transmission electron microscopy (TEM), dynamic light scattering (DLS), nanoparticle tracking analysis (NTA), western blot, enzyme-linked immunosorbent assay (ELISA), and others, respectively. (d) Constituent analysis of urine exosomes is an essential step for predicting DIN. For exosomal protein constituents analysis, western blots, ELISA, tandem mass spectrometry, and LS-MS/MS are commonly used. For exosomal RNA constituents (e.g., mRNA and miRNA) analysis, qPCR, microarrays, and sequencing analysis are commonly used. Standardization of each step is necessary to achieve good reliability and reproducibility in urine exosome tests. In recent years, with the improvement of our knowledge of urine exosomes and the emergence of new technologies, the updated methodology of urine exosomes still requires extensive validation to reach a consensus in the future. In addition, the corresponding detection methods should also be selected based on actual conditions such as urine sample source, quantity, and economic status in clinical applications. Although many scholars have reported their protocol for the isolation or assay of urinary exosomes (11, 78, 100, 103), the current methods for detecting urine exosomes as biomarkers of DIN still have a distance from achieving standardization.

## Summary and outlook

DIN is a severe problem in clinical practice, leading to prolonged hospital stays, increased total treatment costs, and increased mortality (75, 82, 89). Although some drugs have renal toxicity, such as anticancer, antibacterial, antiviral, and non-steroidal anti-inflammatory drugs, their clinical use is still inevitable. In recent years, the nephrotoxicity and safety of Chinese herbs have attracted increasing attention from scholars. The use of Chinese herbs for treating and preventing acute and chronic diseases or promoting health is no longer limited to China and East Asia (80, 92). More and more reports have shown that some Chinese herbs also have apparent toxic effects on the kidneys (e.g., Aristolochia, Thunder god vine, *Glycyrrhiza glabra*, and so on), indicating that the use of natural Chinese herbs does not equate to their clinical medication safety (72, 96). Chinese herbs often contain multiple bioactive components, resulting in a more complex and diverse toxicity mechanism than chemical drugs. How to quickly discontinue or reduce the drug dosage at an appropriate time will be beneficial for maximizing the recovery of renal function. Therefore, in addition to understanding the medicines that have a risk of renal injury, the pathogenesis, and the risk factors of drug-induced nephrotoxicity, real-time effective detection methods are crucial for preventing and reducing the occurrence of renal damage. More and more scholars realize the critical role of urinary exosomes in the event and development of kidney diseases (91, 97). Existing research suggests that urinary exosomes can reflect nephrotoxicity and will further elucidate the molecular mechanisms of DIN, providing new ideas and methods for molecular diagnosis, prognostic judgment, and even treatment of DIN (77). Mishra and colleagues found that urinary exosomes from animal models and patients with diabetic nephropathy (DN) have reno-protective potential (93). Exosome-based drug delivery systems display significant scientific and potential clinical value in the treatment of AKI and other kidney diseases (71, 95, 102). These findings indicate that role switching in urinary exosomes from biomarkers to therapeutic strategies in DIN deserves attention.

Of course, we also realize some challenges that have to be overcome in urinary exosome research on DIN (57). (a) The pathogenesis of drug-induced nephrotoxicity is complex, and the studies of exosomal biomarkers are mostly a description of phenomena, which are still in their infancy, so it is necessary to explore the specific exosomal biomarkers; (b) The enrichment of effective content (e.g., miRNAs and proteins) determines the feasibility of urinary exosomes as biomarkers, but the selection mechanism of exosomes for their content is still unclear; (c) The correlation between changes in exosomal biomarkers and the development of clinically significant acute renal injury is still a great deal to explore, for example, the link between the count of exosomes and eGFR, creatinine levels are still unclear. The research data on exosomes in determining the stage of acute kidney damage linked to drug toxicity is insufficient; (d) The immune cells, bacteria, and yeast in the urogenital tract also secrete exosomes, and some virus particles may be confused with exosomes, so optimization of separation and purification of urinary exosomes are particularly important; (e) So far, the protein assay is still the prioritized method of exosome quantification. However, the protein concentration of exosomes harvested from the existing isolation and purification technologies may not accurately reflect the exosomes’ quantity. Some well-optimized methods still need to be further developed to meet the demand for pure and specific exosomes in the future. Although urinary exosome research is still in the infancy phase and faces some challenges, urinary exosomal biomarkers are a new area for research in the early detection of DIN and have great attraction and application prospects and need further investigation.

## Author contributions

ZZ and YJ had the idea for the review. ZZ and CL wrote the first draft of the manuscript. LW and YY wrote the sections of the manuscript. YJ and XX proofread the manuscript. All the authors contributed to manuscript revision and approved the submitted version.
